# Association Between Dopaminergic Medications and the Evolution of REM Sleep Behavior Disorder in Parkinson's Disease

**DOI:** 10.3389/fneur.2022.880583

**Published:** 2022-06-10

**Authors:** Ruihua Cao, Ruolin Ma, Kai Wang, Panpan Hu

**Affiliations:** ^1^Department of Neurology, The First Affiliated Hospital of Anhui Medical University, Hefei, China; ^2^Hefei Comprehensive National Science Center, Institute of Artificial Intelligence, Hefei, China; ^3^Anhui Province Key Laboratory of Cognition and Neuropsychiatric Disorders, Hefei, China; ^4^Collaborative Innovation Centre of Neuropsychiatric Disorder and Mental Health, Hefei, China

**Keywords:** REM sleep behavior disorder, Parkinson's disease, dopaminergic replacement therapy, longitudinal cohort study, levodopa, dopamine agonist

## Abstract

REM sleep behavior disorder (RBD) is closely associated with Parkinson's disease (PD), however, the influence of dopaminergic replacement therapy (DRT) on the clinical course of RBD in PD remains less understood. The objective of our study is to investigate how DRTs modify the evolution of RBD in a longitudinal cohort study of initially *de novo* PD patients. Four hundred and five drug-naive patients with early-stage PD were included. RBD symptoms were assessed using the 10-item RBD Screening Questionnaire (RBDSQ) at baseline and during the 5-year follow-up. A generalized estimating equation was used to examine predictors of the evolution of RBD symptoms. For patients without baseline pRBD, patients on levodopa treatment showed a greater increase in RBDSQ scores than those not on levodopa treatment, and the increase in RBDSQ scores was significantly correlated with the levodopa-LEDD. Moreover, the changes in RBDSQ scores at a given post-baseline visit were significantly associated with the use of levodopa (OR = 1.875, *p* = 0.008) and the combined use of levodopa and DA (OR = 2.188, *p* = 0.012), as well as the levodopa-LEDD (OR = 1.001, *p* = 0.005) at that visit. The use of DA alone or the DA-LEDD was not a significant predictor of changes in RBDSQ scores. Similarly, a conversion from pRBD negative to pRBD positive was significantly associated with levodopa-LEDD (OR = 1.001, *p* = 0.014) but not DA-LEDD. Together, these finding implicated that the use of levodopa may act as a contributing factor to the increasing prevalence of RBD after the onset of PD, suggesting different mechanisms underlying prodromal RBD and late-onset RBD.

## Introduction

Rapid eye movement (REM) sleep behavior disorder (RBD) is characterized by elaborate motor manifestations related to unpleasant dreams and loss of muscle atonia during REM sleep (1). It is currently the strongest known clinical predictor of the onset of Parkinson's disease (PD) and other synucleinopathies. It has been hypothesized that RBD arises from degeneration or dysfunction of the brain stem circuits that control REM sleep paralysis. This idea fits well with the Braak staging model of PD, which proposes that synucleinopathy pathology starts in the brain stem before ascending rostrally (2).

However, RBD does not necessarily precede PD, and some patients might develop RBD at or after the onset of PD, while in some other patients, RBD symptoms could have disappeared in the first years after motor disturbance onset (3, 4). The presence of RBD has been proposed to be predictive of a more malignant phenotype in PD, including faster cognitive decline (5) and faster motor progression (6, 7). Recently, it has been demonstrated that clinical manifestations of PD may vary depending on not only the presence of RBD but also the timing of RBD onset (8–10), suggesting that there may be two distinct mechanisms behind different timing of RBD and motor symptoms onset of PD.

Emerging evidence has demonstrated the role of dopamine in RBD pathophysiology (11). In a nonhuman primate study, dopaminergic neuronal death caused by a neurotoxin caused heightened muscle activity during REM sleep (12). Neuroimaging studies have revealed nigrostriatal dopaminergic deficits in both isolated RBD (13–16) and RBD associated with PD (17–19). Additionally, there was a significant correlation between increased muscle activity during REM sleep and decreased striatal dopamine transporter levels (14, 16). These studies provide evidence supporting the notion that the genesis of RBD may possibly involve a dopaminergic component. However, the influence of PD and dopaminergic replacement therapy (DRT) on the clinical course of RBD remains less understood.

In the present study, we aimed to investigate the evolution of RBD and how DRTs modify the severity of RBD symptoms in a prospective observational longitudinal cohort study of initially *de novo* PD patients. The correlation between DRTs and RBD evolution may help to understand the mechanism underlying the development of RBD after PD onset, and it may also help provide evidence for the treatment of RBD in PD.

## Materials and Methods

The Parkinson's Progression Markers Initiative (PPMI) is an international, multicenter, prospective cohort study of *de novo* and drug-naive patients with PD. Details of the study have been published elsewhere (20) and are available on the PPMI website (http://www.ppmi-info.org/study-design). Participants meeting the following criteria were recruited between 2010 and 2015: recent diagnosis of PD (<2 years); no past treatment with DRTs; and presenting with at least two of the following: bradykinesia, resting tremor, and rigidity, or with asymmetric resting tremor/bradykinesia at screening. PD diagnosis was confirmed by imaging striatal dopamine transporter deficits at enrollment. All patients underwent a comprehensive longitudinal schedule of clinical assessments. The data used in this study were downloaded from the PPMI database (http://www.ppmi-info.org/data) on 14 February 2020.

### Clinical Evaluation

At baseline, patients were assessed with the Movement Disorders Society-Unified Parkinson's Disease Rating Scale Part III (MDS-UPDRS III), the Hoehn and Yahr (H&Y) scale, the Montreal Cognitive Assessment (MoCA), the 15-item Geriatric Depression Scale (GDS-15) and the State-Trait Anxiety Inventory (STAI).

RBD symptoms were assessed using the 10-item RBD Screening Questionnaire (RBDSQ) at baseline and during the 5-year follow-up. Scores ranged from 0 to 13. A score of 6 or more defined the presence of clinically probable RBD (pRBD). Participants were divided into two subgroups according to baseline RBDSQ scores: the PD-pRBD+ group and the PD-pRBD- group. The evolution of RBD symptoms was defined as the difference between the RBDSQ scores at each follow-up visit and the RBDSQ scores at baseline. A negative difference was considered some mitigation of RBD symptoms, while a positive difference was considered some aggravation of the symptoms.

During follow-up, MDS-UPDRS III scores (“off” state) were assessed, and DRTs used at each visit were recorded. The levodopa equivalent daily doses (LEDDs) were calculated for levodopa and dopamine agonist (DA) separately (21). To preclude the interaction between levodopa and DA, we also divided treatment into four categories at each visit: (1) no DRTs; (2) levodopa only; (3) DA only; and (4) combined levodopa + DA therapy. Other medications that may affect RBD symptoms, including clonazepam, melatonin, antidepressants, and β-blockers, were also recorded.

### Standard Protocol Approvals, Registrations and Patient Consent

The study was approved by the institutional review board at each PPMI site. All patients signed an informed consent form before their participation in the PPMI study.

### Statistical Analysis

Descriptive statistics were used to present baseline characteristics and mean RBDSQ scores and LEDDs over the 5-year follow-up period. Comparisons of changes in RBDSQ scores between patients on different treatments were performed using the Kruskal-Wallis test, with Bonferroni correction for *post hoc* comparisons when appropriate. The generalized estimating equation (GEE), an extension of the generalized linear model for the analysis of longitudinal data, was used to examine predictors of the evolution of RBD. To gain a more complete picture of the longitudinal association between DRTs and the evolution of RBD, we used two models to assess this association. In the first model, we investigated whether the use of DRTs at each post-baseline visit predicted the changes in RBDSQ scores at that same visit. In the second model, we investigated whether the use of DRTs at each post-baseline visit predicted a conversion of clinical RBD diagnosis at the same visit. According to the outcome types, a linear model and a logit model were used. Individual GEE models were created for each clinical predictor of interest, controlling for age, sex, visit, and the use of drugs that may affect RBD symptoms (clonazepam, melatonin, antidepressants, β-blockers) in models. For best fitting model selection, we tested the quasi-likelihood under the quasi-information criterion (QIC) and chose the independent working correlation structure with the smallest QIC. Multicollinearity among parameters was checked by calculating the variance inflation factors (VIF) and ensuring no VIF exceeded 10. All statistical tests were 2-sided. Statistical significance was set at *p* ≤ 0.05. Analyses were conducted with IBM SPSS Statistics (V.22.0).

## Results

### Participant Characteristics

A total of 405 participants with PD (61.6±9.8 years old, 65.4% male) provided baseline and follow-up (at least 1 year) clinical data. Completion rates during follow-up ranged from 96% in the first year to 77% in the fifth year. Mean follow-up period was 4.53 (range: 1–5) years. Baseline demographic and clinical characteristics are provided in Table 1.

**Table 1:** Baseline demographic and clinical characteristics.

**Characteristic**	**Number (%) or mean±SD**
Age, y	61.56 ± 9.78
Male	265 (65.4)
Education, y	15.53 ± 2.94
H&Y stage	1.56 ± 0.51
MDS-UPDRS_III	20.84 ± 8.86
GDS-15	2.32 ± 2.46
STAI	65.19 ± 18.29
STAI-state	32.87 ± 10.25
STAI-trait	32.31 ± 9.46
MoCA	27.10 ± 2.33
Medium follow-up period, y	4.53 ± 1.00

*GDS, 15-item Geriatric Depression Scale; H&Y, Hoehn and Yahr; MDS-UPDRS, Movement Disorder Society–sponsored revision of the Unified Parkinson's Disease Rating Scale; MoCA, Montreal Cognitive Assessment; STAI, State-Trait Anxiety Inventory*.

### Medication and Evolution of RBD Symptoms

At baseline, all patients were not on DRTs; the prevalence rate of pRBD was 24.7%, with a mean RBDSQ score of 4.07±2.66. As the disease progressed, the proportion of patients on DRTs gradually increased to 96.2%, and the prevalence of pRBD increased to 36.9% in the fifth year. The mean RBDSQ scores increased to 4.86±3.19 in the fifth year, which was mainly driven by the PD-pRBD- group. Baseline and follow-up data on RBD assessment and medication status are provided in Table 2.

**Table 2:** Baseline and follow-up data on RBD assessment and medication status.

	**Baseline (*n* = 405)**	**Year 1 (*n* = 389)**	**Year 2 (*n* = 378)**	**Year 3 (*n* = 366)**	**Year 4 (*n* = 342)**	**Year 5 (*n* = 312)**
RBDSQ score	4.07 ± 2.66	4.12 ± 2.80	4.55 ± 2.99	4.58 ± 2.98	4.84 ± 3.19	4.86 ± 3.19
Baseline pRBD+	7.92 ± 1.67	6.59 ± 2.97	7.29 ± 2.74	7.04 ± 2.82	7.45 ± 2.91	7.32 ± 2.82
Baseline pRBD-	2.80 ± 1.43	3.29 ± 2.19	3.66 ± 2.48	3.75 ± 2.54	3.94 ± 2.75	4.08 ± 2.89
pRBD, *n* (%)	100 (24.7)	101 (26.0)	123 (32.5)	124 (33.9)	122 (35.7)	115 (36.9)
On DRTs, n (%)	0	234 (60.2%)	319 (84.4%)	340 (92.9%)	327 (95.6%)	300 (96.2%)
On Levodopa, n (%)	0	88 (22.6)	161 (42.6)	223 (60.9)	249 (72.8)	258 (82.7)
On DA, n (%)	0	95 (24.4)	138 (36.5)	152 (41.5)	149 (43.6)	129 (41.3)
Levodopa-LEDD, mg/d	0	93.55 ± 205.82	192.75 ± 302.84	274.09 ± 329.95	346.48 ± 333.70	439.65 ± 388.06
DA-LEDD, mg/d	0	37.60 ± 78.99	66.59 ± 112.76	78.78 ± 115.72	79.75 ± 117.34	77.69 ± 114.19

*DA, Dopamine agonist; DRT, Dopamine replacement therapy; LEDD, levodopa equivalent daily doses; pRBD, probable REM sleep behavior disorder; RBDSQ, RBD Screening Questionnaire*.

**Figure 1:**
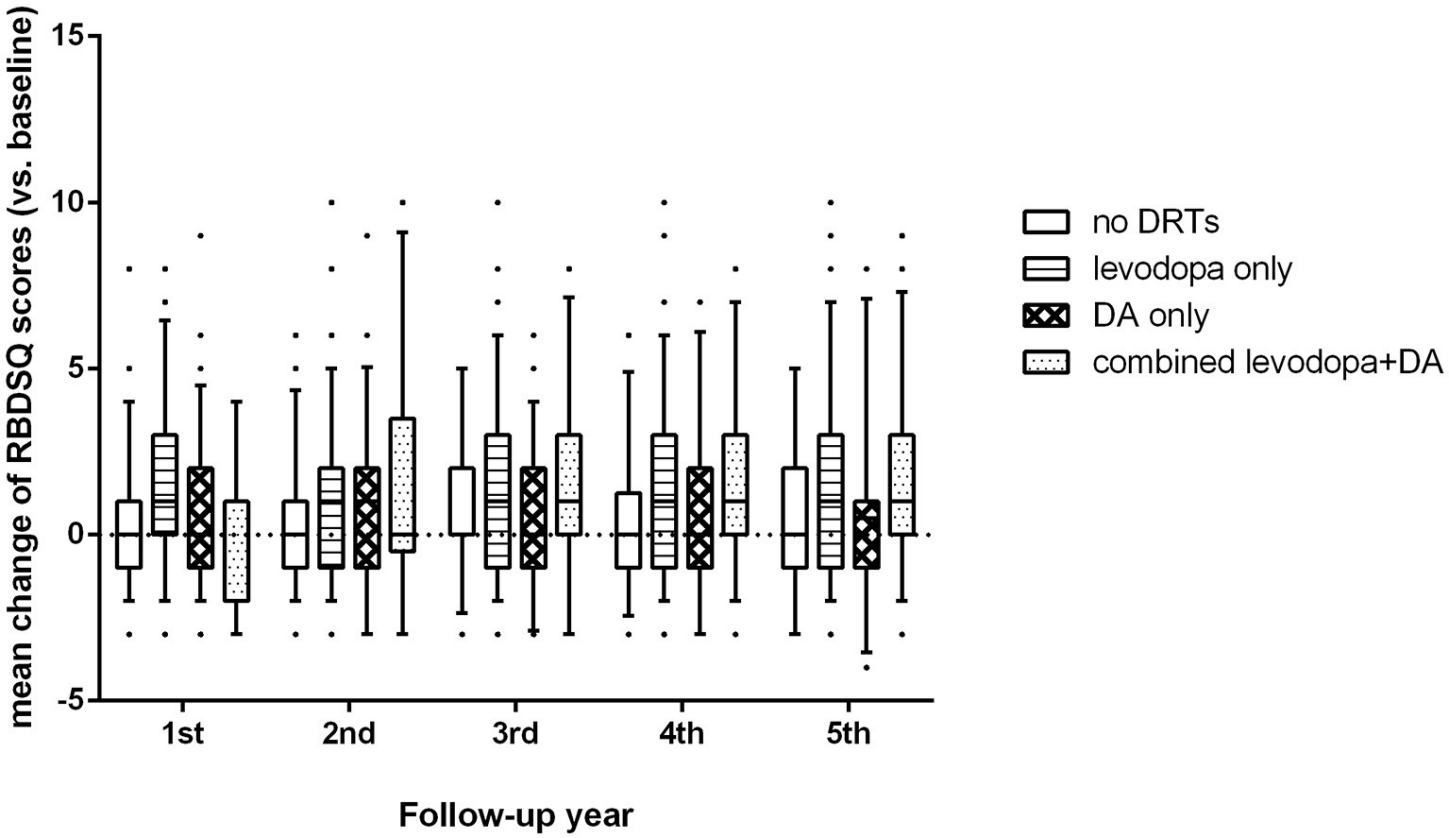
Changes of RBDSQ scores among patients on different treatments.

For the PD-pRBD- group, we found significant differences in changes of RBDSQ scores among patients on different treatments (*p* < 0.001, Figure 1). Specifically, patients on levodopa treatment showed a greater increase in RBDSQ scores than those not on DRTs and those on DA treatment (*p* < 0.001, and *p* = 0.006 respectively). Also, patients on combined levodopa and DA treatment showed a greater increase in RBDSQ scores than those not on DRTs and those on DA treatment (*p* < 0.001, and *p* = 0.005 respectively). No significant differences were found between patients on DA treatment and those not on DRTs. Moreover, the increase in RBDSQ scores was significantly correlated with the levodopa-LEDD but not the DA-LEDD at each visit except for the second year.

### Predictors of the Evolution of RBD Symptoms

For the PD-pRBD- group, the changes in RBDSQ scores at a given post-baseline visit were significantly associated with the use of levodopa alone (OR = 1.875 (1.176 to 2.991), *p* = 0.008) and the combined use of levodopa and DA (OR = 2.188 (1.190 to 4.023), *p* = 0.012) in the linear model (QIC = 7,047.04), as well as levodopa-LEDD (OR = 1.001 (1.000–1.001), *p* = 0.005) at that visit (QIC = 7,061.23). The use of DA alone (OR = 1.134 (0.755–1.704), *p* = 0.544) or the DA-LEDD (OR = 1.000 (0.999–1.002), *p* = 0.622) was not a significant predictor of changes in RBDSQ scores. Similarly, in the logit model (QIC = 1,310.61), a conversion to from pRBD negative to pRBD positive was significantly associated with levodopa-LEDD (OR = 1.001 (1.000–1.001), *p* = 0.014) but not DA-LEDD (OR = 1.001 (0.999–1.002), *p* = 0.574). No multicollinearity detected in these GEE models (VIF < 1.5). Predictors of the evolution of RBD symptoms in the PD-pRBD- group are provided in Table 3. For the PD-pRBD+ group, clinical characteristics and DRTs were not significant predictors of change in RBDSQ scores.

**Table 3:** Predictors of the evolution of RBD symptoms in the PD-pRBD- group.

**Predictor**	**Model of change in RBDSQ scores**		**Model of pRBD conversion**	
	***p***	**OR (95% CI)**	***p***	**OR (95% CI)**
Age	0.973	1.000 (0.979–1.022)	0.520	0.993 (0.972–1.015)
Gender	0.361	0.807 (0.510–1.278)	0.352	0.794 (0.490–1.289)
Melatonin	0.010	2.675 (1.262–5.666)	0.031	2.816 (1.100–7.206)
Benzodiazepine	0.018	4.151 (1.280–13.462)	0.001	3.610 (1.679–7.760)
B-blocker	0.037	1.755 (1.035–2.973)	0.013	1.835 (1.134–2.970)
Antidepressant	0.002	2.155 (1.329–3.494)	0.011	1.922 (1.165–3.170)
Education	0.729	0.988 (0.920–1.060)	0.943	0.997 (0.923–1.077)
Disease duration	0.558	1.009 (0.979–1.039)	0.184	1.023 (0.989–1.059)
H&Y stage	0.217	1.307 (0.855–1.998)	0.429	1.190 (0.774–1.828)
MDS-UPDRS-III_baseline	0.137	1.021 (0.993–1.050)	0.102	1.022 (0.996–1.050)
MDS-UPDRS-III follow-up	0.974	1.000 (0.973–1.027)	0.841	1.002 (0.979–1.026)
GDS	0.379	1.037 (0.956–1.124)	0.261	1.043 (0.970–1.121)
STAI_state	0.242	1.015 (0.990–1.041)	0.532	1.007 (0.986–1.029)
STAI_trait	0.189	1.018 (0.991–1.045)	0.269	1.013 (0.990–1.036)
MoCA	0.436	0.967 (0.888–1.053)	0.460	0.966 (0.880–1.060)
Categories of Medication				
Combined Levodopa and DA	**0.012**	2.188 (1.190–4.023)	0.199	1.533 (0.798–2.945)
DA only	0.544	1.134 (0.755–1.704)	0.638	1.139 (0.662–1.959)
Levodopa only	**0.008**	1.875 (1.176–2.991)	0.133	1.538 (0.903–2.620)
Levodopa-LEDD	**0.005**	1.001 (1.000–1.001)	**0.014**	1.001 (1.000–1.001)
DA-LEDD	0.622	1.000 (0.999–1.002)	0.574	1.001 (0.999–1.002)

*DA, Dopamine agonist; GDS, 15-item Geriatric Depression Scale; H&Y, Hoehn and Yahr; LEDD, levodopa equivalent daily doses; MDS-UPDRS, Movement Disorder Society–sponsored revision of the Unified Parkinson's Disease Rating Scale; MoCA, Montreal Cognitive Assessment; pRBD, probable REM sleep behavior disorder; RBDSQ, RBD Screening Questionnaire; STAI, State-Trait Anxiety Inventory. The bold values indicate the value of P < 0.05*.

## Discussion

In the present study, we used two GEE models to analyze the longitudinal relationship between RBD and DRTs in a prospective observational cohort study of initially *de novo* PD patients. Main results suggest that the association is significant in both models between RBD evolution and levodopa treatment. The use of levodopa may act as a contributing factor to the increasing prevalence of RBD after the onset of PD.

In line with previous studies (3, 22, 23), RBD symptoms evolved during the course of PD. The overall prevalence of RBD and the mean RBDSQ scores increased gradually as PD progressed, which was mainly driven by the baseline pRBD negative patients. In contrast, RBD symptoms improved in some patients with baseline RBD. However, a previous polysomnographic study demonstrated that despite the subjective improvement of RBD symptoms in one-fourth of PD patients with RBD at the 3-year follow-up, all REM sleep without atonia (RSWA) measures increased significantly (24). Another polysomnographic study also reported that all individuals with RBD at baseline were again identified with RBD after 2 years, suggesting that RBD is a robust and stable feature in PD (23). The reason why RBD symptoms improved despite the persistence or worsening of RSWA measures remains unknown.

The main result of our study shows that in a large population of *de novo* and drug-naive PD patients, progression of RBD symptoms was associated with the use of levodopa treatment. Our results were in accordance with previous cross-sectional studies demonstrating that the RBDSQ scores or the frequency of RBD symptoms was positively associated with LEDD of levodopa alone but not of DA (25, 26), and the associations did not account for age, H&Y severity scores (25), or other potentially confounding factors (26). Several longitudinal studies have also investigated the relationship between RBD and DRTs. In line with our findings, a polysomnographic study reported that RBD symptoms and RSWA increased after treatment with levodopa in 15 previously untreated PD patients without RBD, although in the absence of a placebo control group (27). Another study found no significant correlation between the increase in LEDD and RSWA changes in PD patients with RBD (24), which was also in line with our finding that the use of DRTs was not predictive of the evolution of RBD symptoms in the PD-pRBD+ group. Contrary to our findings, one study found no significant correlation between the increase in LEDD and the change in RBD diagnosis (22). There may be some possible explanations for this discrepancy. First, patients in their study were treated at baseline. Second, they did not directly compare the increase in LEDD in patients with different diagnostic outcomes by baseline RBD status, and the patient number in each group was relatively small.

Our results indicate that levodopa therapy may contribute to the later development of RBD in PD. However, as mentioned in a previous cross-sectional study (25), there may be different interpretations of the observed association between the use of levodopa and RBD. That is, the comorbidity of RBD and PD has been shown to predict for a greater severity of motor symptoms, thus the patients with greater increase in RBDSQ scores may show more severe motor symptoms, requiring greater doses of levodopa. However, this interpretation is challenged in several ways. First, neither baseline nor follow-up MDS-UPDRS III scores predicted the evolution of RBD symptoms, suggesting that more severe pathology underlying the higher PD severity may not sufficient to explain the greater increase in RBDSQ scores in these patients. Second, it is baseline pRBD that has been shown to predict for more rapid progression of motor symptoms (7), while patients were all baseline pRBD negative in the models demonstrating the observed association in the present study.

The role of dopamine mechanisms in RBD pathogenesis has not been fully understood. Medullary and/or pontine REM sleep-related structures are responsible for triggering motor suppression during normal REM sleep and are closely connected to midbrain dopaminergic pathways and may therefore be affected by an imbalance in dopamine levels (11, 28, 29). The fact that changes of RBDSQ scores were associated with the use of levodopa but not the use of DA may point to a role of D1 receptors activation in facilitating the pathogenesis of RBD, as most current dopamine agonists selectively activate D2 and D3, but not D1 receptors. Previous evidence has shown that D1 receptor agonists or antagonists modulate REM sleep (30, 31). Rotigotine, the unique non-ergot-derived dopamine agonist that can activate D1 receptors has been shown to partially improve RBD-related symptoms (32). Moreover, the dopamine D1 receptor agonist SKF38393 has also been shown to restored REM sleep to baseline values in MPTP-treated monkeys (33).

The relationship between DRTs and the later development of RBD indicate that there may be different mechanisms underlying early-onset and late-onset RBD in PD. Specifically, the development of RBD during the prodromal phase may arise from degeneration or dysfunction of the brain stem circuits, as the Braak staging model suggests (34). Moreover, previous literature finds that a large fraction of idiopathic RBD cases still have nigrostriatal dopamine innervation within normal limits (35, 36), suggesting that the dopaminergic system may have little role in early-onset RBD. In contrast, the development of late-onset RBD may rely on dopaminergic modulation to a larger extent. This may partly explain the different clinical manifestations of PD by the timing of RBD onset; for example, it has been reported that patients with late onset RBD showed a higher disease stage, took higher dopaminergic therapy, and had a longer disease duration (8).

There are some limitations in our study. First, RBD diagnosis was based on a questionnaire instead of polysomnography; however, the RBDSQ is a validated screening tool for RBD both in the general population and in PD patients, and it has been used as a measure of RBD severity, as surface EMG activity was significantly associated with RBDSQ scores (37). Second, as previously reported (22, 38), repetitive RBD assessment could have increased patient and caregiver awareness of mild symptoms, resulting in an increase in RBD cases at follow-up. This limitation might be balanced by the fact that it probably existed for all patients regardless of the treatment strategies. Last, there were other medications known to relieve or induce RBD symptoms. We tried to eliminate their influence by regressing out these factors.

In summary, our data found that RBD symptoms evolved during the course of PD, and the evolution was associated with the use of levodopa. We speculated that the dopaminergic system may play a more important role in the development of RBD after PD onset than in the development of RBD before PD onset.

## Data Availability Statement

The data analyzed in this study was obtained from the Parkinson's Progression Markers Initiative (PPMI), the following licenses/restrictions apply: Investigators seeking access to PPMI data must sign the Data Use Agreement, submit an Online Application and comply with the study Publications Policy. Requests to access these datasets should be directed to PPMI, https://ida.loni.usc.edu/collaboration/access/appLicense.jsp.

## Ethics Statement

The studies involving human participants were reviewed and approved by the Parkinson's Progression Markers Initiative (PPMI). The patients/participants provided their written informed consent to participate in this study.

## Author Contributions

RC and RM contributed to the study concept and design, writing of the manuscript, statistical analysis, and interpretation of data. PH and KW contributed to the study concept and design, interpretation of data, and revision of the manuscript. All authors contributed to the article and approved the submitted version.

## Funding

For up-to-date information on the study, visit www.ppmi-info.org. PPMI—a public-private partnership—is funded by the Michael J. Fox Foundation for Parkinson's Research and funding partners, including AbbVie, Allergan, Avid Radiopharmaceuticals, Biogen, BioLegend, Bristol-Myers Squibb, Celgene, Covance, GE Healthcare, Genentech, GlaxoSmithKline, Lilly, Lundbeck, Merck, Meso Scale Discovery, Pfizer, Piramal, Prevail, Roche, Sanofi Genzyme, Servier, Takeda, TEVA, UCB, Verily, Voyager, and Golub Capital.

## Conflict of Interest

The authors declare that the research was conducted in the absence of any commercial or financial relationships that could be construed as a potential conflict of interest.

## Publisher's Note

All claims expressed in this article are solely those of the authors and do not necessarily represent those of their affiliated organizations, or those of the publisher, the editors and the reviewers. Any product that may be evaluated in this article, or claim that may be made by its manufacturer, is not guaranteed or endorsed by the publisher.
